# Reduction in clinically important deterioration in chronic obstructive pulmonary disease with aclidinium/formoterol

**DOI:** 10.1186/s12931-017-0583-0

**Published:** 2017-05-30

**Authors:** Dave Singh, Anthony D. D’Urzo, Ferran Chuecos, Anna Muñoz, Esther Garcia Gil

**Affiliations:** 1 The University of Manchester, Medicines Evaluation Unit, Centre for Respiratory and Allergy Medicine, University Hospital of South Manchester Foundation Trust, Manchester, M23 9QZ UK; 2 Department of Family and Community Medicine, Faculty of Medicine, University of Toronto, Toronto, Canada; 3 AstraZeneca PLC, Barcelona, Spain; 4 Former employee of AstraZeneca PLC, Barcelona, Spain

**Keywords:** COPD, LABA, LAMA, Bronchodilation, Chronic respiratory disease

## Abstract

**Background:**

‘Clinically important deterioration’ (CID) is a composite endpoint measuring worsening of the key clinical features of chronic obstructive pulmonary disease (COPD), namely lung function, patient-reported outcomes, and exacerbations. ACLIFORM and AUGMENT were two 24-week, randomized, double-blind, phase III studies assessing twice-daily (BID) aclidinium bromide (AB) 400 μg/formoterol fumarate (FF) 12 μg. This pooled post-hoc analysis assessed the effects of AB/FF 400/12 μg on both first and sustained CID events versus placebo and monotherapies in patients with moderate to severe COPD.

**Methods:**

A first CID event was defined as the occurrence of a moderate/severe exacerbation or the worsening from baseline in ≥1 of the following: trough forced expiratory volume in 1 second (FEV_1_; ≥100 mL), Transition Dyspnea Index (TDI) focal score (≥1 unit), or St George’s Respiratory Questionnaire (SGRQ) total score (≥4 units). A ‘sustained’ CID was defined as a worsening maintained at all subsequent visits from appearance to week 24 or a moderate/severe exacerbation at any time. CID events were assessed at three visits (weeks 4, 12, and 24); trough FEV_1_ was also measured at weeks 1 and 18.

**Results:**

AB/FF 400/12 μg reduced the risk of a first CID event by 45% versus placebo (hazard ratio [HR] 0.55, *p* < 0.001), 18% versus FF 12 μg (HR 0.82, *p* < 0.01), and 15% versus AB 400 μg (HR 0.85, *p* < 0.05). Similarly, AB/FF 400/12 μg reduced the risk of a sustained CID event by 48% versus placebo (HR 0.52, *p* < 0.001) and 22% versus FF 12 μg (HR 0.78, *p* < 0.01). AB/FF 400/12 μg reduced the risk of a first or sustained CID event for all four components versus placebo (trough FEV_1_ and TDI, first and sustained CID, all *p* < 0.001; SGRQ first CID *p* < 0.001; SGRQ sustained CID, *p* < 0.01; exacerbations first and sustained CID, both *p* < 0.05) and TDI and SGRQ versus FF 12 μg (TDI, first and sustained CID both *p* < 0.05; SGRQ first CID *p* < 0.01), and SGRQ versus AB 400 μg (first CID, *p* < 0.05).

**Conclusions:**

AB/FF 400/12 μg BID may provide greater airway stability and fewer exacerbations or deteriorations in lung function, health status, or dyspnea compared with placebo or monotherapies.

**Trial registration:**

Clinicaltrials.gov NCT01462942 (ACLIFORM); registered 26 October 2011.

Clinicaltrials.gov NCT01437397 (AUGMENT); registered 19 September 2011.

**Electronic supplementary material:**

The online version of this article (doi:10.1186/s12931-017-0583-0) contains supplementary material, which is available to authorized users.

## Background

Chronic obstructive pulmonary disease (COPD) is a chronic and heterogeneous disease where dyspnea is a prevailing clinical presentation, among others, that often responds favorably to bronchodilator therapy [1, 2]. In recent years, the benefits of dual bronchodilator therapy (a combination of a long-acting β_2_-agonist [LABA] and long-acting muscarinic antagonist [LAMA]) versus monotherapy in COPD have been well documented and have included outcomes such as lung function, quality of life, dyspnea, exercise tolerance, and exacerbation events [3–10]. Such efficacy studies typically focus on how treatment interventions improve outcomes that reflect the multicomponent nature of COPD and also emphasize the importance of achieving current control and the reduction of important future events such as exacerbations, hospitalizations, and mortality. In many of these studies, there is often much less focus on describing those patients whose clinical status deteriorates during the course of study participation. To date, there are few studies that describe composite endpoints that focus on patients who experience a clinical worsening of COPD control [11]. This information is likely to be useful for our understanding of treatment effects among patients with COPD with variable clinical presentations.

Recently, a new composite endpoint has been introduced that measures worsening of the key clinical features of COPD, namely lung function and symptoms. This has been called ‘clinically important deterioration’ (CID) [11] and uses not only the occurrence of moderate to severe exacerbations (defined by the requirement for oral steroids and/or antibiotics), but also the minimal clinically important differences (MCIDs) for forced expiratory volume in 1 second (FEV_1_) and patient-reported outcomes (PROs).

The CID endpoint may be used to identify either temporary fluctuations in disease state that recover, or a more sustained worsening in disease. A previous post-hoc analysis has shown that the combination bronchodilator umeclidinium/vilanterol reduced the risk of first and sustained CID events, compared with placebo and long-acting bronchodilator monotherapy [11]. In this study, a CID was defined as a deterioration in trough FEV_1_ of ≥100 mL, or a ≥4-unit increase in St George’s Respiratory Questionnaire (SGRQ) total score, or an on-treatment moderate to severe COPD exacerbation; a CID was considered sustained when it occurred on ≥2 consecutive visits 4 weeks apart or for ≥50% of all available subsequent visits. The results indicated that the majority of the study population suffered with a CID and the analysis suggested that an important attribute of LABA/LAMA combinations is to stabilize disease characteristics and prevent CID events.

ACLIFORM and AUGMENT were two 24-week, randomized, double-blind, phase III studies that successfully demonstrated the efficacy and safety of twice-daily aclidinium bromide 400 μg (a LAMA) and formoterol fumarate 12 μg (a LABA) combined treatment in patients with moderate to severe COPD [4, 6].

The use of CID as a composite endpoint in clinical trials is in its infancy and it is important to generate more analysis of clinical trial data using CID events so that we can fully understand its potential value. Furthermore, the definitions of first CID and sustained CID events should be further explored and, if appropriate, refined. The aim of the current analysis was to generate more information on the CID composite endpoint using the components previously used, while adding the Transition Dyspnea Index (TDI). We also evaluated sustained CID events by including events with a longer duration than previously considered. We performed a post-hoc analysis of pooled data from the ACLIFORM and AUGMENT studies to assess the ability of aclidinium/formoterol 400/12 μg to reduce the risk of both first and sustained CID events, and their components over 24 weeks, versus placebo and monotherapies in patients with moderate to severe, stable COPD.

## Methods

### Study design

ACLIFORM (NCT01462942) and AUGMENT (NCT01437397) were two 24-week, randomized, double-blind, placebo- and active-controlled parallel-group phase III studies in patients with moderate to severe COPD [4, 6]. Both studies were conducted in accordance with the International Conference on Harmonisation/Good Clinical Practice guidelines and the Declaration of Helsinki. The protocol for each study was approved by the relevant participating center’s Institutional Review Board and all patients provided written informed consent before participating in any study procedure.

Patients were randomized 2:2:2:2:1 (ACLIFORM) or 1:1:1:1:1 (AUGMENT) to receive aclidinium bromide (AB) 400 μg/formoterol fumarate (FF) 12 μg, AB 400 μg/FF 6 μg, AB 400 μg monotherapy, FF 12 μg monotherapy, or placebo, twice daily for up to 24 weeks. Of the two doses of AB/FF, only the 400/12 μg dose was investigated in this pooled post-hoc analysis. All treatments were administered using the same multidose dry powder inhaler (Genuair™/Pressair®^1^).

### Patients

Key inclusion criteria for both ACLIFORM and AUGMENT included male or female patients aged ≥40 years, current or former smoker, and a diagnosis of stable moderate to severe COPD (post-bronchodilator FEV_1_/forced vital capacity ratio <70% and post-bronchodilator FEV_1_ ≥ 30% and <80% predicted). Key exclusion criteria included history or current diagnosis of asthma, respiratory infection or COPD exacerbation ≤6 weeks (≤3 months if hospitalized for exacerbations) prior to screening, clinically significant respiratory conditions other than COPD, and clinically significant cardiovascular conditions.

Patients were permitted to use inhaled salbutamol (100 μg/puff) as reliever medication as needed; however, long-acting bronchodilators other than the study drug were not permitted during the study. Treatment with inhaled corticosteroids (ICS) was permitted, provided treatment was stable for ≥4 weeks prior to screening.

### Clinically important deterioration

For the purposes of this post-hoc analysis, a CID event was defined as ≥1 of the following outcomes:

- Deterioration of ≥100 mL from baseline in pre-dose (trough) FEV_1_
- Deterioration of ≥1 unit in TDI focal score
- Deterioration of ≥4 units from baseline in SGRQ total score
- Occurrence of a moderate/severe COPD exacerbation.

CID events were recorded at three study visits during the treatment period (weeks 4, 12, and 24); trough FEV_1_ was measured at two additional study visits (week 1 and week 18). A sustained CID event was defined as deteriorations in trough FEV_1_, SGRQ, and/or TDI that were maintained at all subsequent visits from appearance to week 24, or any moderate/severe exacerbation. CID events were subsequently stratified by COPD severity at baseline (moderate and severe COPD; ≥50% and <50% predicted post-bronchodilator FEV_1_, respectively) and by those patients who were receiving treatment with a LABA and / or LAMA prior to the study and those who were not.

A sensitivity analysis comparing all visits with common visits was performed for the CID composite, along with an analysis of the individual components of the composite endpoint. An assessment of the impact of stratifying patients by ICS use, the presence of symptoms at baseline, and whether or not patients had received previous treatment before screening visit was performed to assess the impact of these variables on the risk of CID events.

### Statistical analyses

The intent-to-treat (ITT) population was used for efficacy analyses and was defined as all randomized patients who took at least one dose of study medication and had a baseline and at least one post-baseline FEV_1_ assessment. The risk of a CID event over time was analyzed using a Cox-Proportional Hazard model with study treatment group as a covariate.

## Results

### Patients

The overall pooled population from ACLIFORM and AUGMENT included 3394 patients in the ITT population. This post-hoc analysis included data from 2680 patients receiving either AB/FF 400/12 μg, AB 400 μg, FF 12 μg or placebo; patients receiving AB/FF 400/6 μg were not included. Patient demographics and baseline characteristics of the study population were similar across all treatment groups (Table 1).

**Table 1:** Patient demographics and baseline characteristics (ITT population)

	AB/FF 400/12 μg (*N* = 720)	AB 400 μg (*N* = 720)	FF 12 μg (*N* = 715)	Placebo (*N* = 525)
Mean age, years	63.4	63.7	63.5	63.7
Male, *n* (%)	429 (59.6)	442 (61.4)	423 (59.2)	313 (59.6)
White, *n* (%)	672 (93.3)	675 (93.8)	672 (94.0)	499 (95.0)
Current smoker, *n* (%)	354 (49.2)	351 (48.8)	350 (49.0)	263 (50.1)
Cigarette smoking, mean pack-years (SD)	46.6 (25.1)	44.9 (23.7)	45.8 (22.4)	49.2 (26.5)
Mean FEV_1_, L (SD)	1.387 (0.524)	1.376 (0.519)	1.397 (0.510)	1.382 (0.550)
Post-bronchodilator percent of predicted FEV_1_, % (SD)	53.9 (13.2)	53.3 (13.2)	54.2 (13.1)	53.5 (13.4)
COPD severity				
Moderate, *n* (%)^a^	418 (58.1)	411 (57.1)	436 (61.0)	293 (55.8)
Severe, *n* (%)^b^	301 (41.8)	306 (42.5)	278 (38.9)	231 (44.0)
Mean number of exacerbations in previous 12 months (SD)	0.5 (0.9)	0.5 (0.8)	0.4 (0.8)	0.3 (0.7)
Mean BDI focal score (SD)	6.4 (2.1)	6.5 (2.1)	6.4 (2.2)	6.5 (2.2)
Mean SGRQ total score (SD)	46.8 (17.4)	46.1 (17.3)	45.5 (18.0)	45.5 (17.8)
Previous treatment with a LABA and / or LAMA, n (%)^c^	355 (49.3)	371 (51.5)	362 (50.6)	254 (48.4)
Baseline ICS use, *n* (%)	259 (36.0)	255 (35.4)	244 (34.1)	183 (34.9)

^a^Patients with ≥50% predicted post-bronchodilator FEV_1_

^b^Patients with <50% predicted post-bronchodilator FEV_1_

^c^Regardless of ICS use

*AB* aclidinium bromide, *BDI* Baseline Dyspnea Index, *COPD* chronic obstructive pulmonary disease, *FEV*_*1*_ forced expiratory volume in 1 second, *FF* formoterol fumarate, *ICS* inhaled corticosteroid, *ITT* intent-to-treat, *LABA * long-acting β﻿_2_-agonist, *LAMA* long-acting muscarinic antagonist, *SD* standard deviation, *SGRQ* St George’s Respiratory Questionnaire

### First CID events

**Fig. 1:**
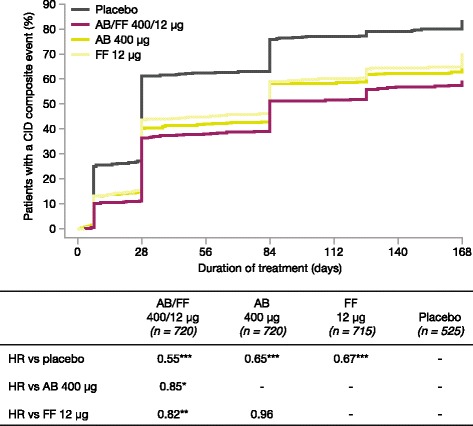
Analysis of time to first CID event (ITT population). *p < 0.05, **p < 0.01, ***p < 0.001. The risk of a first CID event was analyzed using a Cox-Proportional Hazard model including study, treatment group, and smoking status as covariates. AB, aclidinium bromide; CID, clinically important deterioration; FF, formoterol fumarate; HR, hazard ratio

The percentage of patients with a first CID event ranged from 57.8% in AB/FF 400/12 μg to 74.9% in placebo (Fig. 1). AB/FF 400/12 μg reduced the risk of a first CID event over 24 weeks by 45% compared with placebo (hazard ratio [HR] 0.55, *p* < 0.001), by 18% versus FF 12 μg (HR 0.82, *p* < 0.01), and by 15% versus AB 400 μg (HR 0.85, *p* < 0.05; Fig. 1). FF 12 μg and AB 400 μg also significantly reduced the risk of a first CID event compared with placebo (both *p* < 0.001).

**Fig. 2:**
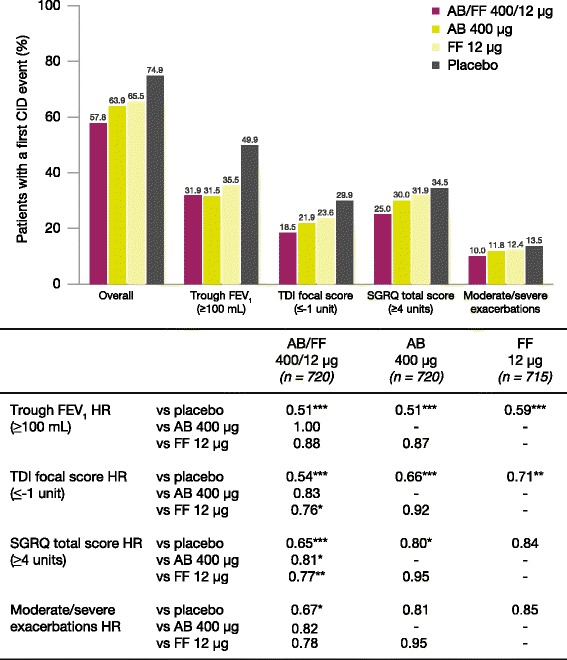
Analysis of first CID events by individual components over 24 weeks (ITT population). *p < 0.05, **p < 0.01, ***p < 0.001. The risk of a first CID event was analyzed using a Cox-Proportional Hazard model including study, treatment group, and smoking status as covariates. AB, aclidinium bromide; CID, clinically important deterioration; FEV_1_, forced expiratory volume in 1 second; FF, formoterol fumarate; HR, hazard ratio; ITT, intent-to-treat; SGRQ, St George’s Respiratory Questionnaire; TDI, Transition Dyspnea Index

Results for the individual CID components are summarized in Fig. 2. For AB/FF 400/12 μg and both monotherapies, there was a significant reduction in the risk of a first CID event in FEV_1_ versus placebo (all *p* < 0.001) and no differences between active treatments. For TDI, AB/FF 400/12 μg and both monotherapies demonstrated a significant reduction in the risk of a first CID event versus placebo (*p* < 0.001 for AB/FF 400/12 μg and AB 400 μg; *p* < 0.01 for FF 12 μg), while there was also a significant reduction in risk for AB/FF 400/12 μg compared with FF 12 μg (*p* < 0.05). There was a significant reduction in the risk of a first CID event in SGRQ, with both AB/FF 400/12 μg and AB 400 μg versus placebo (*p* < 0.001 and *p* < 0.05, respectively) and for AB/FF 400/12 μg versus both FF 12 μg (*p* < 0.01) and AB 400 μg (*p* < 0.05). For exacerbations, only AB/FF 400/12 μg demonstrated a statistically significant reduction in the risk of a first CID event compared with placebo (*p* < 0.05).

### Sustained CID events

**Fig. 3:**
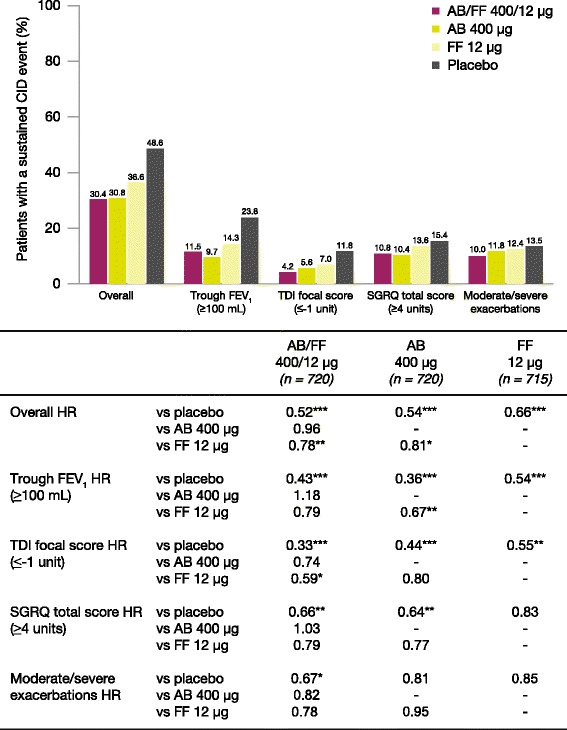
Analysis of sustained CID events overall and by individual components over 24 weeks (ITT population). *p < 0.05, **p < 0.01, ***p < 0.001 HR vs placebo. The risk of a sustained CID event was analyzed using a Cox-Proportional Hazard model including study, treatment group, and smoking status as covariates. AB, aclidinium bromide; CID, clinically important deterioration; FEV_1_, forced expiratory volume in 1 second; FF, formoterol fumarate; HR, hazard ratio; ITT, intent-to-treat; SGRQ, St George’s Respiratory Questionnaire; TDI, Transition Dyspnea Index

The percentage of patients with a sustained CID event ranged from 30.4% in AB/FF 400/12 μg to 48.6% in placebo (Fig. 3). AB/FF 400/12 μg reduced the risk of a sustained CID event over 24 weeks by 48% compared with placebo (HR 0.52, *p* < 0.001) and by 22% versus FF 12 μg (HR 0.78, *p* < 0.01; Fig. 3). Differences in the risk of a sustained CID event for AB/FF 400/12 μg versus AB 400 μg were not significant. AB 400 μg reduced the risk of a sustained CID event versus FF 12 μg (HR 0.81, *p* < 0.05).

Results for the individual sustained CID components are summarized in Fig. 3. For AB/FF 400/12 μg and both monotherapies, there was a significant reduction in the risk of a sustained CID event in FEV_1_ versus placebo (all *p* < 0.001). For TDI, AB/FF 400/12 μg and both monotherapies demonstrated a significant reduction in the risk of a sustained CID event versus placebo (*p* < 0.001 for AB/FF 400/12 μg and AB 400 μg; *p* < 0.01 for FF 12 μg), and there was a significant reduction in risk with AB/FF 400/12 μg compared with FF 12 μg (*p* < 0.05). Both AB/FF 400/12 μg and AB 400 μg showed a significant reduction in the risk of a sustained CID event in SGRQ versus placebo (both *p* < 0.01). For exacerbations, only AB/FF 400/12 μg demonstrated a statistically significant reduction in the risk of a sustained CID event compared with placebo (*p* < 0.05).

### Analysis by previous treatment with a LABA or LAMA

**Fig. 4:**
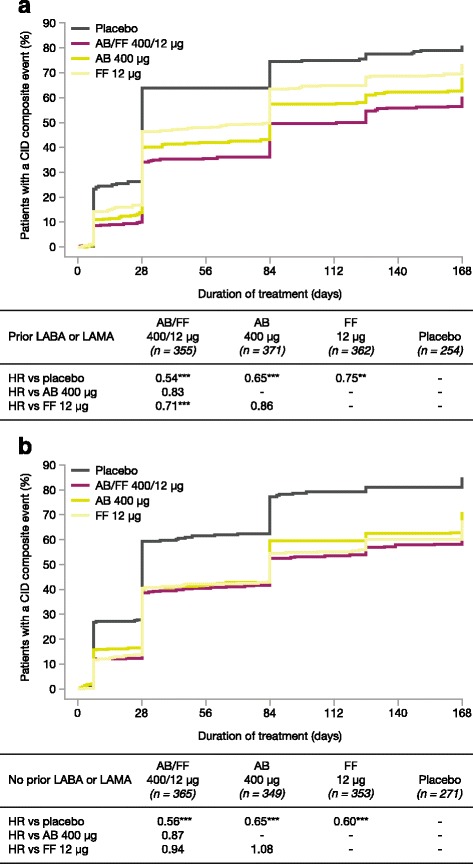
Analysis of time to first CID event in patients who (**a**) received treatment with a LABA and / or LAMA and (**b**) did not receive treatment with a LABA and / or LAMA prior to the study (ITT population). *p < 0.05, **p < 0.01, ***p < 0.001. The risk of a first CID event was analyzed using a Cox-Proportional Hazard model including study, treatment group, and smoking status as covariates. AB, aclidinium bromide; CID, clinically important deterioration; FF, formoterol fumarate; HR, hazard ratio; ITT, intent-to-treat; LABA, long-acting β_2_-agonist; LAMA, long-acting muscarinic antagonist

For patients who were receiving treatment with a LABA or LAMA prior to the study, AB/FF 400/12 μg reduced the risk of a first CID event versus placebo and FF 12 μg (both *p* < 0.001) but not versus AB 400 μg (Fig. 4a). AB 400 μg and FF 12 μg also significantly reduced the risk of a first CID event compared with placebo (*p* < 0.001 and *p* < 0.01, respectively). In those patients who were not receiving treatment with a LABA or LAMA prior to the study, AB/FF 400/12 μg reduced the risk of a first CID event versus placebo only (*p* < 0.001; Fig. 4b). AB 400 μg and FF 12 μg also significantly reduced the risk of a first CID event versus placebo (both *p* < 0.001). Similar results were obtained for the risk of sustained CID events (Additional file 1: Table S1).

### Analysis by COPD severity at baseline

**Fig. 5:**
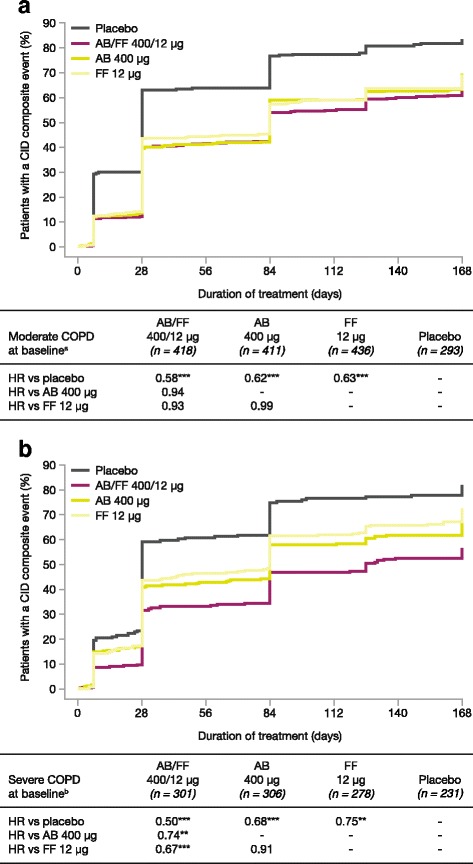
Analysis of time to first CID event in patients with (**a**) moderate and (**b**) severe COPD at baseline (ITT population). *p < 0.05, **p < 0.01, ***p < 0.001. ^a^Patients with ≥50% predicted post-bronchodilator FEV_1_; ^b^Patients with <50% predicted post-bronchodilator FEV_1_. The risk of a first CID event was analyzed using a Cox-Proportional Hazard model including study, treatment group, and smoking status as covariates. AB, aclidinium bromide; CID, clinically important deterioration; FF, formoterol fumarate; HR, hazard ratio; ITT, intent-to-treat

For patients with moderate COPD (≥50% predicted post-bronchodilator FEV_1_), AB/FF 400/12 μg reduced the risk of a first CID event versus placebo (*p* < 0.001) with no differences in risk of CID events between active treatments (Fig. 5a). For patients with severe COPD (<50% predicted post-bronchodilator FEV_1_), AB/FF 400/12 μg reduced the risk of a first CID event compared with placebo (*p* < 0.001), AB 400 μg (*p* < 0.01) and FF 12 μg (*p* < 0.001; Fig. 5b). AB 400 μg and FF 12 μg also significantly reduced the risk of a first CID event versus placebo (*p* < 0.001 and *p* < 0.01, respectively). Similar results were obtained for the risk of sustained CID events (Additional file 1: Table S2).

### Sensitivity analysis of all study visits versus common study visits

The outcome of the sensitivity analysis of first and sustained CID events, comparing all study visits (weeks 1, 4, 12, 18, and 24) with common study visits (weeks 4, 12, and 24 only), can be seen in Additional file 1: Tables S3 and S4, respectively. The risk of both first and sustained CID events with AB/FF 400/12 μg versus placebo and monotherapy was similar between all visits and common visits.

### Analysis according to number of CID components achieved

An analysis based on the number of CID endpoint components achieved is shown in Additional file 1: Table S5. Overall, 48.7%, 34.3%, 14.9%, and 2.1% of patients had a CID event based on one, two, three, or four components of the composite, respectively. For placebo and FF 12 μg, there was a numerical increase in the number of patients who achieved two (approximately 39%) rather than one component (approximately 38% and 45%, respectively), compared with AB/FF 400/12 μg and AB 400 μg (approximately 30% with two components and 55% with one component).

### Analysis by ICS, symptoms, and previous treatment

There were few differences when analyzing first and sustained CID events stratified by baseline ICS use, symptoms (assessed using Evaluating Respiratory Symptoms or Baseline Dyspnea Index), and whether or not patients had received previous treatment (Additional file 1: Figure S1).

## Discussion

In this pooled post-hoc analysis of patients from the phase III ACLIFORM and AUGMENT studies, we report that a deterioration in clinical status, as described by both first CID and sustained CID events, was commonly observed in patients with COPD. AB/FF 400/12 μg reduced the risk of a first CID event versus placebo and both monotherapies. In addition, there was a reduction in the risk of a sustained CID event versus placebo and FF 12 μg. Interestingly, analysis of the individual components of the composite showed that there were statistically significant differences between AB/FF 400/12 μg and the monotherapies for TDI and SGRQ, but not FEV_1_. This suggests that the differentiation of AB/FF 400/12 μg treatment from monotherapy in this analysis is not due to effects on FEV_1_, but rather the prevention of symptom worsening.

Previously, analysis of CID has not included TDI as one of the components of the composite endpoint. TDI is a well-recognized PRO for measuring improvements in dyspnea, either on a group mean basis or individual responder analysis. We now demonstrate that TDI also identifies individuals with symptom worsening. The inclusion of TDI in the CID composite is supported by our results showing that TDI differentiated dual bronchodilator treatment from both FF 12 µg and placebo. Interestingly, FEV_1_ was not able to differentiate the effects of dual bronchodilator treatment from monotherapy, unlike the PROs TDI and SGRQ. It is well known that changes in FEV_1_ are often poorly correlated with changes in symptoms at an individual level, and the analysis reported here suggests a benefit of dual bronchodilation that is not observed when measuring FEV_1_.

Deteriorations in FEV_1_ were the most commonly reported CID component, followed by deteriorations in SGRQ and TDI. A potential criticism of events that are FEV_1_ deteriorations only is that they are not accompanied by PRO change, and so their clinical importance is unclear. However, we observed that many CID events occurred with more than one component being met; for example, approximately 62% of CID events in patients with COPD who received placebo occurred with more than one component achieved. We suggest that CID events with more than one component achieved are more likely to be clinically important events. Exacerbations were present during only 10–14% of CID events, indicating that the majority of CID events with more than one component endpoint being met were due to a combination of FEV_1_ and PRO endpoints. Of note, the risk of a CID event and all components except lung function was higher for monotherapies versus AB/FF 400/12 μg, indicating that differences here may be driven by PRO endpoints.

There was a shift towards two components being present with less effective treatments, i.e. FF 12 μg and placebo. It appears that less effective treatments have more CID events, which are also different in nature and more likely to be clinically important due to a shift in the number of components present. We added TDI to the endpoints previously used in CID analysis, and we suggest that this is a useful change, making it easier to evaluate whether FEV_1_ changes are accompanied by PRO changes during a CID event.

At the start of the run-in period, all patients had their maintenance treatment withdrawn and were to remain stable for the duration of the run-in period before they could undergo randomization. It is possible, however, that patients receiving treatment with either a LABA or a LAMA prior to the study may have been randomized to placebo and therefore a reduction in the previous level of treatment may have contributed to any deterioration observed. Stratifying the results by prior treatment revealed that AB/FF 400/12 μg was more effective in reducing the risk of a CID event than FF 12 µg and placebo in patients who had received a LABA and / or LAMA prior to the study compared with those who had not. Long acting bronchodilator withdrawal may have contributed to these CID events in these patients, but nevertheless these results provide support for the concept that dual bronchodilator therapy provides better protection against CID events than long acting bronchodilator monotherapy.

When considering the effects of treatment in patients when data were stratified by moderate and severe airflow obstruction at baseline, AB/FF 400/12 μg provided greater benefits in reducing the risk of a CID event compared with monotherapies and placebo in patients with severe COPD than patients with moderate COPD. This subgroup analysis again provides information on the subgroups of patients more likely to benefit from dual bronchodilator therapy in terms of CID prevention.

The definition that we used for a sustained CID event ensured that these were deteriorations in FEV_1_ and/or PROs that patients did not recover from during the study duration, or a moderate to severe exacerbation. The nature of these sustained CID events in the absence of a diagnosis of exacerbation is unclear. It is possible that some of these events were prolonged, unreported exacerbations; it is known that some unreported exacerbations are associated with longer-term impact [12]. An alternative possibility is that these sustained events represent disease progression. Further investigation is needed to understand the nature of sustained CID events, and whether changes in individual components could be more indicative of unreported exacerbations, or an indication of disease progression. Moreover, analysis of CID events may enable the detection of a high rate of deterioration in a patient population that typically displays a low exacerbation rate.

There are some methodological points to consider in CID composite endpoint analysis. The thresholds of ≥100 mL in trough FEV_1_, ≥1 unit in TDI focal score, and ≥4 units in SGRQ total score used were based on recognized MCID for changes in lung function and health status [13–15]. It may be useful, however, to assess whether these are the most appropriate thresholds for understanding long-term outcomes and whether this varies with the patient population studied. Stratifying the results by ICS use, whether or not patients were symptomatic, and whether or not patients had received prior treatment for COPD, did not reveal any differences between treatment groups. It is not clear whether these findings might be influenced by the size of the populations of the various subgroups analyzed; larger studies of longer duration may be required to clarify this issue. Additionally, clinical trials often collect more lung function information than other endpoints such as PROs. A sensitivity analysis of first CID and sustained CID events at all study visits compared with common visits revealed no overall differences between treatments, indicating that inclusion of the additional trough FEV_1_ measurements (weeks 1 and 18) did not have any impact on either outcome. One must also consider that the post-hoc nature of the CID assessments reported here could be considered a limitation. In addition, it is worth noting that the relatively short 24-week duration of the studies is of limited use when analyzing clinical deterioration and further studies of longer duration and prospective in nature will be of benefit.

The finding that a substantial number of patients experienced first and sustained CID events highlights the heterogeneity related to therapeutic responsiveness across treatment interventions and a care gap that requires further study. Further investigations may also focus on developing prediction models that can be used to identify patients who are most likely to be protected against clinical deterioration with conventional treatments like those described here.

## Conclusions

In this pooled post-hoc analysis, treatment with AB/FF 400/12 μg twice daily reduced the risk of a first CID event versus placebo, AB 400 μg, and FF 12 μg. Furthermore, reductions in the risk of a sustained CID event were observed for AB/FF 400/12 μg compared with placebo and FF 12 μg. Reductions in the risk of a CID event in the individual components of the composite were also observed. Overall, these results suggest that AB/FF 400/12 μg may provide greater airway stability and therefore fewer deteriorations in lung function, health status, dyspnea, and the occurrence of exacerbations compared with placebo or monotherapies.

## Additional file

Additional file 1: Sensitivity analysis of the risk of CID events for common visits, characterization of patients according to CID endpoints achieved and analysis of the risk of CID events over 24 weeks, stratified by ICS use, previous treatment, and symptoms. (DOCX 289 kb)

## Abbreviations

AB: Aclidinium bromide
BDI: Baseline dyspnea index
BID: Twice daily
CID: Clinically important deterioration
COPD: Chronic obstructive pulmonary disease
FEV_1_: Forced expiratory volume in 1 second
FF: Formoterol fumarate
HR: Hazard ratio
ICS: Inhaled corticosteroids
ITT: Intent-to-treat
LABA: Long-acting β_2_-agonist
LAMA: Long-acting muscarinic antagonist
MCID: Minimal clinically important difference
PRO: Patient-reported outcome
SD: Standard deviation
SGRQ: St George’s respiratory questionnaire
TDI: Transition dyspnea index

## Footnotes

1 Registered trademarks of the AstraZeneca group of companies; for use within the USA as Pressair® and Genuair™ within all other licensed territories.
