# Supplementary material for: Reduction in clinically important deterioration in chronic obstructive pulmonary disease with aclidinium/formoterol
Source: Respir Res. 2017 May 30;18:106. doi: 10.1186/s12931-017-0583-0 (PMC5450266; doi:10.1186/s12931-017-0583-0)
Supplement: Additional file 1: — Sensitivity analysis of the risk of CID events for common visits, characterization of patients according to CID endpoints achieved and analysis of the risk of CID events over 24 weeks, stratified by ICS use, previous treatment, and symptoms. (DOCX 289 kb) [file 12931_2017_583_MOESM1_ESM.docx]

**Additional File 1**

**Supplementary Table 1.** Analysis of sustained CID events by previous treatment with a LABA and / or LAMA (ITT population)

| **Prior LABA or LAMA** | **AB/FF  400/12 µg** | **AB  400 µg** | **FF  12 µg** | **Placebo** |
| --- | --- | --- | --- | --- |
| Yes, n | 355 | 371 | 362 | 254 |
| HR vs placebo | 0.55*** | 0.48*** | 0.74* | - |
| HR vs AB 400 µg | 1.16 | - | - | - |
| HR vs FF 12 µg | 0.75* | 0.65*** | - | - |
| No, n | 365 | 349 | 353 | 271 |
| HR vs placebo | 0.48*** | 0.61*** | 0.58*** | - |
| HR vs AB 400 µg | 0.78 | - | - | - |
| HR vs FF 12 µg | 0.82 | 1.05 | - | - |

*p<0.05, ***p<0.001
The risk of a sustained CID event was analyzed using a Cox-Proportional Hazard model including study, treatment group, and smoking status as covariates
AB, aclidinium bromide; CID, clinically important deterioration; FF, formoterol fumarate; HR, hazard ratio; LABA, long-acting β_2_-agonist; LAMA, long-acting muscarinic antagonist

**Supplementary Table 2.** Analysis of sustained CID events by COPD severity at baseline (ITT population)

| **COPD at baseline** | **AB/FF  400/12 µg** | **AB  400 µg** | **FF  12 µg** | **Placebo** |
| --- | --- | --- | --- | --- |
| Moderate^a^, n | 418 | 411 | 436 | 293 |
| HR vs placebo | 0.57*** | 0.50*** | 0.61*** | - |
| HR vs AB 400 µg | 1.13 | - | - | - |
| HR vs FF 12 µg | 0.94 | 0.83 | - | - |
| Severe^b^, n | 301 | 306 | 278 | 231 |
| HR vs placebo | 0.44*** | 0.60*** | 0.77* | - |
| HR vs AB 400 µg | 0.74* | - | - | - |
| HR vs FF 12 µg | 0.58*** | 0.78 | - | - |

*p<0.05, ***p<0.001
^a^Patients with ≥50% predicted post-bronchodilator FEV_1_; ^b^Patients with <50%predicted post-bronchodilator FEV_1_
The risk of a sustained CID event was analyzed using a Cox-Proportional Hazard model including study, treatment group, and smoking status as covariates
AB, aclidinium bromide; CID, clinically important deterioration; COPD, chronic obstructive pulmonary disease; FF, formoterol fumarate; HR, hazard ratio; ITT population

**Supplementary Table 3.** Sensitivity analysis of the risk of first CID events for common visits over 24 weeks (ITT population)

|  | **AB/FF 400/12 µg (n = 720)** | **AB 400 µg (n = 720)** | **FF 12 µg (n = 715)** | **Placebo**  **(n = 525)** |
| --- | --- | --- | --- | --- |
| Percentage of patients with  ≥1 CID during the study^a^ | 54.6 | 60.4 | 61.7 | 70.3 |
| HR vs placebo | 0.61*** | 0.71*** | 0.74*** | - |
| HR vs AB 400 µg | 0.85* | - | - | - |
| HR vs FF 12 µg | 0.82** | 0.97 | - | - |

*p<0.05, **p<0.01, ***p<0.001

^a^Weeks 4, 12, and 24

The risk of a first CID event was analyzed using a Cox-Proportional Hazard model including study, treatment group, and smoking status as covariates

AB, aclidinium bromide; CID, clinically important deterioration; FF, formoterol fumarate; HR, hazard ratio; ITT, intent-to-treat

**Supplementary Table 4.** Sensitivity analysis of the risk of sustained CID events for common visits over 24 weeks (ITT population)

|  | **AB/FF 400/12 µg (n = 720)** | **AB 400 µg (n = 720)** | **FF 12 µg (n = 715)** | **Placebo**  **(n = 525)** |
| --- | --- | --- | --- | --- |
| Percentage of patients with  ≥1 sustained CID during the study^a^ | 19.7 | 22.5 | 27.1 | 37.5 |
| HR vs placebo | 0.47*** | 0.54*** | 0.66*** | - |
| HR vs AB 400 µg | 0.86 | - | - | - |
| HR vs FF 12 µg | 0.71** | 0.82 | - | - |

**p<0.01, ***p<0.001

^a^Weeks 4, 12, and 24

The risk of a sustained CID event was analyzed using a Cox-Proportional Hazard model including study, treatment group, and smoking status as covariates

AB, aclidinium bromide; CID, clinically important deterioration; FF, formoterol fumarate; HR, hazard ratio; ITT, intent-to-treat

**Supplementary Table 5.** Characterization of patients according to CID endpoints achieved (ITT population)

| **Number of CIDs, n (%)** | **AB/FF 400/12 µg (n = 462)** | **AB 400 µg (n = 511)** | **FF 12 µg (n = 519)** | **Placebo  (n = 444)** | **Total  (n = 1936)** |
| --- | --- | --- | --- | --- | --- |
| 1^a^ | 258 (55.8) | 281 (55.0) | 234 (45.1) | 170 (38.3) | 943 (48.7) |
| 2^b^ | 138 (29.9) | 152 (29.7) | 202 (38.9) | 173 (39.0) | 665 (34.3) |
| 3^c^ | 60 (13.0) | 67 (13.1) | 74 (14.3) | 87 (19.6) | 288 (14.9) |
| 4^d^ | 6 (1.3) | 11 (2.2) | 9 (1.7) | 14 (3.2) | 40 (2.1) |

^a^Deterioration in any CID component of trough FEV_1_, TDI focal score, SGRQ total score, or exacerbation

^b^Any two CIDs of trough FEV_1_ + TDI focal score, trough FEV_1_ + SGRQ total score, trough FEV_1_ + exacerbation, TDI focal score + SGRQ total score, TDI focal score + exacerbation, or SGRQ total score + exacerbation

^c^Any three CIDs of trough FEV_1_ + TDI focal score + SGRQ total score, FEV_1_ + TDI focal score + exacerbation, trough FEV_1_ + SGRQ total score + exacerbation, TDI focal score + SGRQ total score + exacerbation

^d^All four exacerbations, trough FEV_1_ + TDI focal score + SGRQ total score + exacerbation

AB, aclidinium bromide; CID, clinically important deterioration; FEV_1_, forced expiratory volume in 1 second; FF formoterol fumarate; ITT, intent-to-treat; SGRQ, St George’s Respiratory Questionnaire; TDI, Transition Dyspnea Index

**Supplementary Table 6.** List of ethics committees

| Ethikkommission der Medizinischen, Universität Graz, Auenbruggerplatz 2, Graz, 8036, Austria |
| --- |
| Ethisch Comité UZA, Wilrijkstraat 10, Edegem, 2650, Belgium |
| Ethics Committee for Multicenter Trials (ECMT), 5, ‘Sveta Nedelya’, Square 1000 Sofia, Bulgaria |
| Agency for Medicinal Product and Medical Devices of Croatia, Central Ethics Committee, Ksaverska cesta 4 Zagreb, 10000, Croatia |
| Multicentricka eticka komise Fakultni nemocnice u sv. Anny v Brne, Vystavni 17/19, Brno, 656 19, Czech Republic |
| De Videnskabsetiske Komitéer for Region Hovedstaden, Kongens Vænge 2, 3400, Hillerød, Denmark |
| Keski-Suomen sairaanhoitopiiri, Eettinen toimikunta, Sairaanhoitopiirin toimisto Rak. 6/2, Keskussairaalantie 19, 40620 Jyväskylä, Finland |
| Comité de Protection des Personnes Sud Ouest et Outre Mer III, Place Amélie Raba-Léon, Groupe Hospitalier Pellegrin – Service de Pharmacologie Clinique, Bât 1 A, Bordeaux Cedex, 33076, France |
| Landesärztekammer Rheinland-Pfalz, Place Amélie Raba-Léon, Groupe Hospitalier Pellegrin – Service de Pharmacologie Clinique, Bât 1 A, Bordeaux cedex, 33076, Germany |
| Egészségügyi Tudományos Tanács Klinikai Farmakológiai Etikai Bizottsága, Arany J. u. 6–8, Budapest, H-1051, Hungary |
| Comitato Etico Locale per la Sperim. Clin. dei Medicinali dell'Az. Osp.ra Univ.ria Senese di Siena, c/o Farmacia AOUS Viale Bracci, Siena, 53100, Italy |
| METC Catharina Ziekenhuis, Michelangelolaan 2, Eindhoven, 5623 EJ, Netherlands |
| Komisja Bioetyczna przy Instytucie Gruzlicy I Chorob Pluc, ul. Plocka 26, Warszawa, 01–138, Poland |
| Comisia Nationala de Etica, Str. Aviator Sanatescu Nr. 48, Bucuresti, Sector 1, 011478, Romania |
| Ethical Council at the MoH of RF, 3 Rakhmanovsky Pereulok, Moscow, 127994, Russia |
| Fakultná nemocnica s poliklinikou F.D. Roosevelta, Nám. L. Svobodu 1, 975 17 Banská Bystrica, Slovakia |
| CEIC Hospital Universitario Puerta de Hierro Majadahonda, Planta 1ª, Pasillo unidades, administrativas de servicios, c/ Manuel de Falla, 1, Majadahonda, 28222, Spain |
| Regionala Etikprövningsnämnden I Lund, Box 133, Östra Vallgatan 14/Östervångsvägen 1, Lund, 22100, Sweden |
| Central Ethics Commission of the Ministry of Health of Ukraine, 5, Narodnogo Opolchennya St., Kyiv, 03680, Ukraine |
| NRES Committee North, West-Greater Liverpool, Central, 3^rd^ Floor, Barlow House, 4 Minshull Street, Manchester, M1 3DZ, United Kingdom |

**Supplementary Figure legend**

**Supplementary Figure 1.** Analysis of the risk of CID events over 24 weeks, stratified by a) ICS use, b) previous treatment, c) symptoms defined by E-RS, and d) symptoms defined by BDI (ITT population)


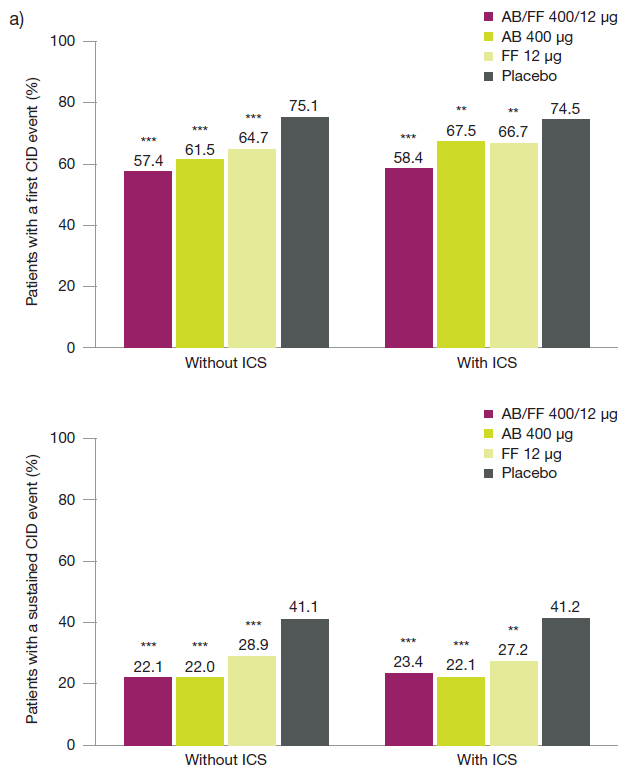


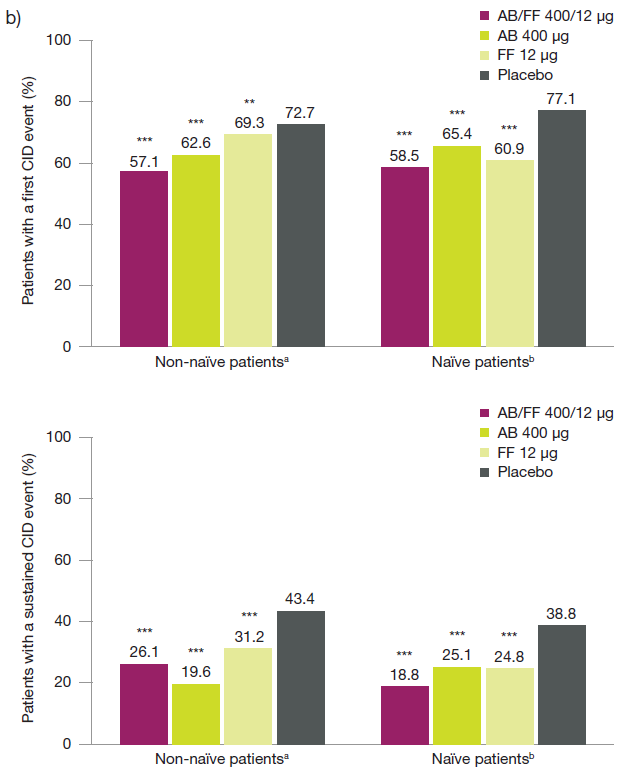


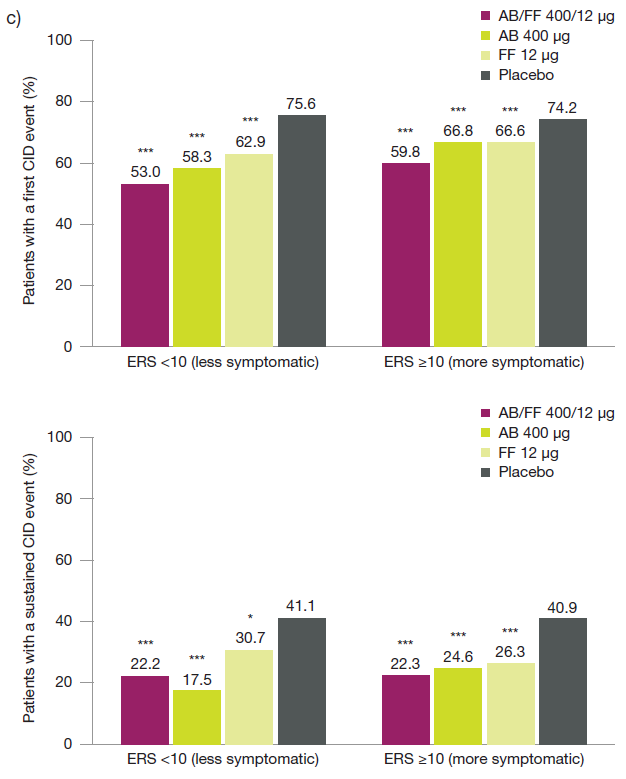


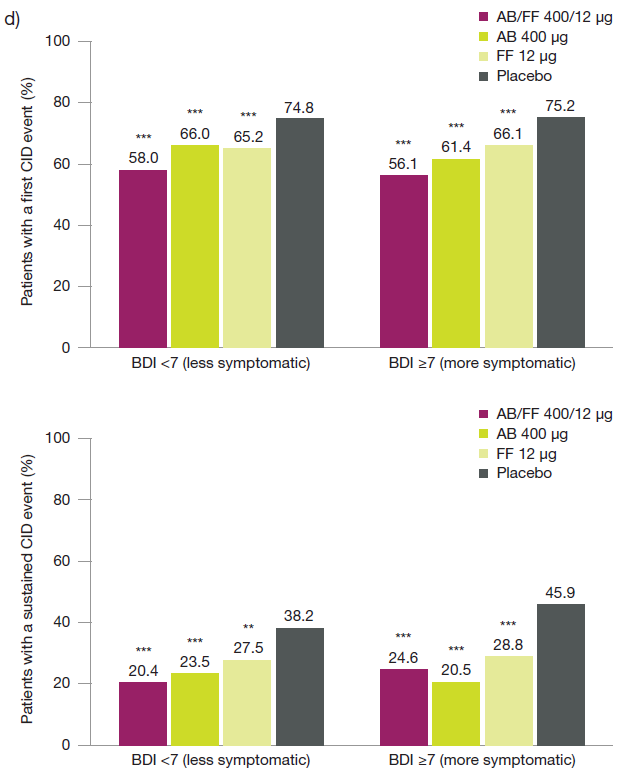


*p<0.05, **p<0.01, ***p<0.001 HR vs placebo

^a^Defined as patients previously treated with LABAs and/or LAMAs, LABA/ICS combinations, or LABA/LAMA/ICS combinations or xanthines

^b^Defined as patients who were not previously treated with SABAs and/or SAMAs or with other medications (ICS, leukotriene antagonists, systemic corticosteroids, oxygen or influenza vaccine) not in accordance with GOLD guidelines [1].

Risk of first or sustained CID events and each individual component were analyzed using a Cox-Proportional Hazard model including study, treatment group, and smoking status as covariates

AB, aclidinium bromide; BDI, baseline dyspnea index; CID, clinically important deterioration; E-RS, Evaluating Respiratory Symptoms; FF, formoterol fumarate; GOLD, Global initiative for chronic Obstructive Lung Disease; HR, hazard ratio; ICS, inhaled corticosteroid; ITT, intent-to-treat; LABA, long-acting β_2_-agonist; LAMA, long-acting muscarinic antagonist; SABA, short-acting β_2_-agonist; SAMA, short-acting muscarinic antagonist

**References**

1. Global Initiative for Chronic Obstructive Lung Disease. Global strategy for the diagnosis, management, and prevention of chronic obstructive pulmonary disease 2017. [[http://goldcopd.org/gold-2017-global-strategy-diagnosis-management-prevention-copd/]](http://goldcopd.org/gold-2017-global-strategy-diagnosis-management-prevention-copd/%5d). Accessed 1 Mar 2017.
